# Extracellular Synthesis of Luminescent CdS Quantum Dots Using Plant Cell Culture

**DOI:** 10.1186/s11671-016-1314-z

**Published:** 2016-02-24

**Authors:** Mariya N. Borovaya, Olga M. Burlaka, Antonina P. Naumenko, Yaroslav B. Blume, Alla I. Yemets

**Affiliations:** Department of Genomics and Molecular Biotechnology, Institute of Food Biotechnology and Genomics, National Academy of Sciences of Ukraine, Osypovskogo Str, 2a, 04123 Kyiv, Ukraine; Faculty of Physics, Taras Shevchenko National University, Acad. Glushkova Ave, 4, 03022 Kyiv, Ukraine

## Abstract

The present study describes a novel method for preparation of water-soluble CdS quantum dots, using bright yellow-2 (BY-2) cell suspension culture. Acting as a stabilizing and capping agent, the suspension cell culture mediates the formation of CdS nanoparticles. These semiconductor nanoparticles were determined by means of an UV-visible spectrophotometer, photoluminescence, high-resolution transmission electron microscopy (HRTEM), and XRD. Followed by the electron diffraction analysis of a selected area, transmission electron microscopy indicated the formation of spherical, crystalline CdS ranging in diameter from 3 to 7 nm and showed wurtzite CdS quantum dots. In the present work, the toxic effect of synthesized CdS quantum dots on *Nicotiana tabacum* protoplasts as a very sensitive model was under study. The results of this research revealed that biologically synthesized CdS nanoparticles in low concentrations did not induce any toxic effects.

## Background

At present, semiconductor nanoparticles are a focus of wide-ranging studies. It has been shown that the size of these particles determines their properties [1, 2]. CdS is a II-VI semiconductor with the direct band-gap energy of 2.42 eV [3]. At the nanometer scale, this material is found at an intermediate level between atomic, molecular, and bulk, revealing new physical properties [2]. The macroscopic physical properties of CdS nanoparticles and nanocrystals measuring less than 10 nm conform to quantum mechanics rules. The spatial restriction of a nanoparticle affects the wavelength of electrons, which is reduced compared with the bulk. This effect is referred as the quantum-confinement or quantum-size effect [2]. CdS has a great potential for usage in photochemical catalysis, solar cells, nonlinear optical materials, various luminescent devices, probes for exploring DNA structures, and fluorescent probes for peptide detection [4]. The physico-chemical methods of a synthesis are commonly used to produce cadmium sulfide nanoparticles. For example, CdS nanoparticles can be prepared by the hydrothermal-microemulsion method [5], wet chemical coprecipitation method [6], and ultrasonic irradiation [7]. Such methods require a special laboratory setup and they are time-consuming. Thus, a simple and novel «green» method of semiconductor nanoparticle synthesis is a matter of considerable scientific interest [8] today.

As a matter of fact, successful preparation and characterization of CdS quantum dots produced by bacteria and fungi have been discussed in our early studies [9–11]. However, using plants and plant extracts for the CdS biosynthesis is of great interest that can be explained by being environmentally friendly, the fast growth of a plant biomass, and the low cost of initial material. We showed recently that CdS quantum dots could be successfully obtained using plant hairy root culture [12]. Simultaneously with our investigation, two independent groups of scientists demonstrated efficient methods of the biosynthesis of cadmium sulfide quantum dots, using the leaf extract of *Asparagus racemosus* [13] and banana peel extract [14].

The biosynthesis of CdS quantum dots by cell suspension culture provides a novel approach, which is non-toxic, easily reproducible, and not time-consuming. Obtained semiconductor nanoparticles acquire unique physical properties that are due to a reduction in their size. Therefore, in the present research, we aimed to synthesize water-soluble CdS quantum dots, using cell suspension culture of *Nicotiana tabacum* (cv. bright yellow-2 (BY-2)). In this work, we demonstrated the specific optical and structural-morphological features of produced semiconductor quantum dots. Moreover, this study assesses the toxic effect of produced CdS quantum dots on *N. tabacum* protoplasts.

## Methods

### Biological Synthesis of CdS Quantum Dots

The biological synthesis of CdS nanoparticles was carried out using cell suspension culture of *N. tabacum* L. cv. bright yellow-2 (BY-2). The plant cell culture was grown in a liquid MS medium [15] at 28 °C on the rotary shaker at 135 rpm during 7 days. In order to produce cadmium sulfide quantum dots, the suspension culture of *N. tabacum* (BY-2) was filtered through filter paper under aseptic conditions to remove the culture medium. The resultant cell biomass was diluted in a conical flask (100 mL) with sterile, deionized water and incubated at 28 °C on a rotary shaker (135 rpm) during 24 h. Then, 2 mL of 0.025 М CdSO_4_ and 500 μL of 0.5 M Na_2_S water solutions (Sigma-Aldrich, USA, 98 % purity) were poured into a 100-mL flask with BY-2 cells with total cell mass of 613 mg. Right after that, we observed the formation of a homogeneous bright yellow solution of CdS nanoparticles. To remove the cell biomass, the resultant solution was centrifuged at 5000 rpm for 10 min (MiniSpin Eppendorf, USA). The plant matrix without adding CdSO_4_ and Na_2_S solutions also was centrifuged as has been described above, and the resultant supernatant was used as a control for further analysis. The freshly prepared solution of CdS quantum dots was passed through the nitrocellulose filter Millipore (USA) with pores of 0.45 μm in diameter. Then, 10 mL of produced CdS quantum dots were used for optical and structural-morphological studies.

### Luminescence of CdS Quantum Dots

Luminescence spectra were measured at room temperature, using the serial spectrophotometer Cary Eclipse (Varian Inc., Agilent Technologies, USA). The highest resolution of this spectrophotometer amounted to 1.5 nm and was determined by the apparatus function and the smallest width of a gap. Selected spectrum gap width for the measurement was 5 nm. The accuracy of wavelength recording was 0.05 nm, and the accuracy of intensity determination did not exceed 1 %. Device software provided the correction of the spectra by taking into account a sensitivity curve in consideration of the spectral sensitivity of a multiplier photocell used in a fluorometer. Standard quartz cuvettes (1 × 1 × 3 cm^3^) were used for spectral measurements. To determinate wavelengths correctly, a spectral array was separated into four components. A spectral division was carried out with the aid of an automatic software for spectroscopy (PeakFit 4.11).

### UV-Visible Absorption Spectrophotometry

The absorption spectra of CdS nanoparticles were measured by the spectrophotometer Specord UV-VIS Analytik Jena AG (Germany). The absorption spectra of samples were recorded in standard quartz 10-mm cuvettes (transmission range 170 ÷ 1000 nm). According to the protocol, the accuracy of recording of wave numbers was 20 cm^−1^. Owing to digital processing and random factors, however, the actual experimental accuracy was 80 cm^−1^. Optical density was determined within the accuracy of up to 1 % of the optical scale length, the optical density ranging from 0 to 1.4. The spectrum recorded by the chart recorder Specord UV vis was analyzed by a computer scanner and converted into the image as a jpeg file. Then, the resultant file was processed by the software package GetData converting the spectrum data into the digital format of a dat-file. The numerical data were processed by the software Origin Pro 8.0. A spectral division was carried out using PeakFit 4.11.

### High-Resolution Transmission Electron Microscopy

CdS quantum dots were investigated by means of the electron microscope JEOL JEM-2100F (Japan) with the accelerating voltage of 200 kV. Each sample was dispersed ultrasonically to separate individual particles, and some drops of suspension deposited onto carbon-coated copper grids. Experimental material was precipitated by evaporation and used for further studies.

### Electron Diffraction Analysis

Electron diffraction patterns of the CdS quantum dots, which deposited on the carbon-coated copper grid, were obtained by means of the microscope JEOL JEM-2100F at electron beam energy $\mathsf{E}=\mathsf{100}$ kеV (wavelength of electrons $\lambda =\mathsf{0.012}$ nm). Localization of a beam on the sample was 200 nm.

### Toxic Effect of Produced CdS Quantum Dots on *N. tabacum* Protoplasts

In order to study the toxic effect of synthesized CdS quantum dots on cells, *N. tabacum* protoplasts were used as a model object. To this end, plants were grown in vitro at 24 °C and for a photoperiod of 16 h. Protoplasts were isolated from the aseptic *N. tabacum* plants by enzymatic degradation of a cell wall as described in [16]. Concentration of the stock solution of CdS quantum dots was 0.193 mg/mL. In order to test the toxicity of synthesized quantum dots, we diluted the quantum dots stock solution twofold (0.097 mg/mL), fourfold (0.049 mg/mL), eightfold (0.025 mg/mL), 16-fold (0.012 mg/mL), 32-fold (0.006 mg/mL), 64-fold (0.003 mg/mL), and 128-fold (0.002 mg/mL). Then CdS quantum dots were added to the freshly isolated protoplasts of *N. tabacum* and treated during 24 h.

Survivability of the protoplasts was investigated by means of the luminescent microscope Axioskop 40 (“Carl Zeiss”, Germany). Computer processing of micrographs was performed using the software AxioVision LE 4.8.2.0 (“Carl Zeiss MicroImaging GmbH”, Germany, 2010). We determined a survival rate as the ratio of undamaged protoplasts to the total number of protoplasts. A count of the protoplasts was made by the Goryaev counting chamber. Statistical data processing was done using the Student’s *t* test [17].

## Results and Discussion

### Optical Spectrum Analysis

**Fig. 1:**
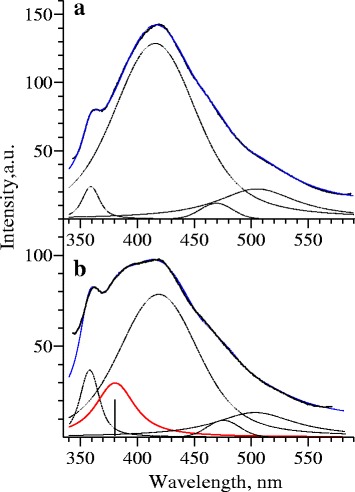
Luminescence spectrum of *N. tabacum* matrix (**a**); *blue line*—synthesized contour of plant matrix (the sum of spectral components), *black line*—experimental contour, *dashed lines*—the spectral components of *N. tabacum* luminescence spectrum. *Red line*—an additional component, corresponding to CdS quantum dots luminescence (**b**)

**Fig. 2:**
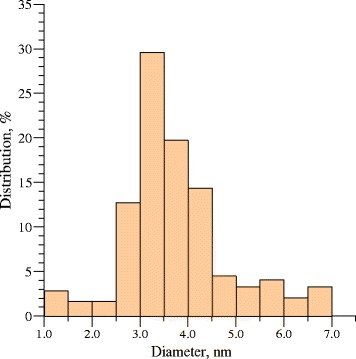
CdS nanoparticles size distribution histogram determined from HRTEM micrographs. Total number of nanoparticles was 243

The luminescence spectrum of the *N. tabacum* matrix, which was used as a control, and the luminescence spectrum of produced CdS quantum dots are shown in Fig. 1a, b. These luminescence spectra were separated into four spectral components that are described by Voigt functions with different width values of Gaussian and Lorenz components (dashed lines in Fig. 1a). The luminescence spectrum of CdS quantum dots differs from the spectrum of the *N. tabacum* matrix due to the presence of a shoulder at the wavelengths of 380–400 nm. The spectrum of CdS nanoparticles was also separated as described above, but an additional line was allocated. This line corresponds to the indicated shoulder (red line in Fig. 1b). The synthesized spectrum is shown in Fig. 1b as a blue line. It has been found that a synthesized contour is almost identical to the experimental spectrum. Thus, we can affirm that luminescence of CdS quantum dots is described by a spectral component marked as a red line. The wavelength of this maximum is 381 nm (3.25 eV). It is believed that at the wavelength λ = 381 nm (3.25 eV), the luminescent peak corresponds to transitions 1_*se*_-1_*sh*_ between dimensional quantization levels in CdS nanoparticles with a different diameter [18]. Using a relationship between energy of the optical transition 1_*se*_-1_*sh*_ and the diameter of the CdS nanoparticles established in [18], we determined that photon energy at 3.25 eV corresponds to nanoparticles of 3.4–3.5 nm in diameter. The results of research were in a good agreement with high-resolution transmission electron microscopy (HRTEM) data. According to the HRTEM, maximum-sized nanoparticle distribution corresponds to 3.0–4.0 nm (Fig. 2).

**Fig. 3:**
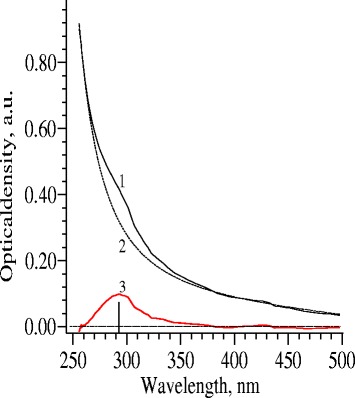
Absorption spectrum of CdS quantum dots. (*1*) experimental contour, (*2*) monotonically decreased background, (*3*) line corresponding to the absorbance of CdS quntum dots. Maximum at the wavelength 292 nm

The absorption spectrum also indicates small-sized CdS nanoparticles (Fig. 3). In particular, a clear absorption peak can be observed at a wavelength of 292 nm. This maximum is obtained by the allocation of a monotonically decreasing background (dashed line in Fig. 3) from an experimental contour (solid line). Also noticeable is that the distinct absorption peak at 295 nm was shown earlier in the study [13] for biologically produced CdS quantum dots. Our previous optical investigations of the CdS quantum dots correlate with the present data. In particular, we reliably established that the absorption band of the CdS quantum dots shifted in a shortwave region [9, 12]. Luminescence peaks depend heavily on the size of nanoparticles. As compared with our previous results, the particle size of CdS quantum dots produced by *Pleurotus* mycelium and *Linaria* root extract averaged 4.0–5.5 nm and 5.5–7.0 nm, respectively, that correlated with optical measurements indicating distinct luminescent peaks at 431, 462, and 486 nm [10, 11], as well as 462 and 500 nm for larger nanoparticles in [12]. Consequently, we obtained more homogeneous and smaller CdS quantum dots in this study.

### High Resolution Transmission Electron Microscopy Analysis

**Fig. 4:**
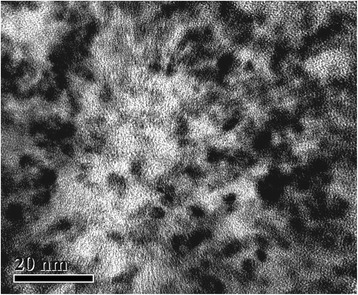
HRTEM image of synthesized CdS quantum dots; *scale bar* indicates 20 nm

**Fig. 5:**
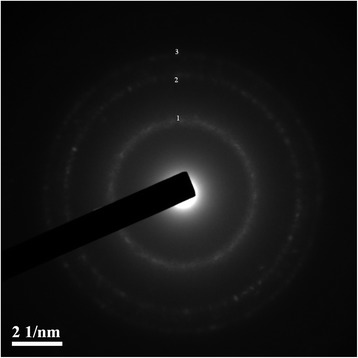
Electron diffraction pattern of synthesized CdS quantum dots

**Fig. 6:**
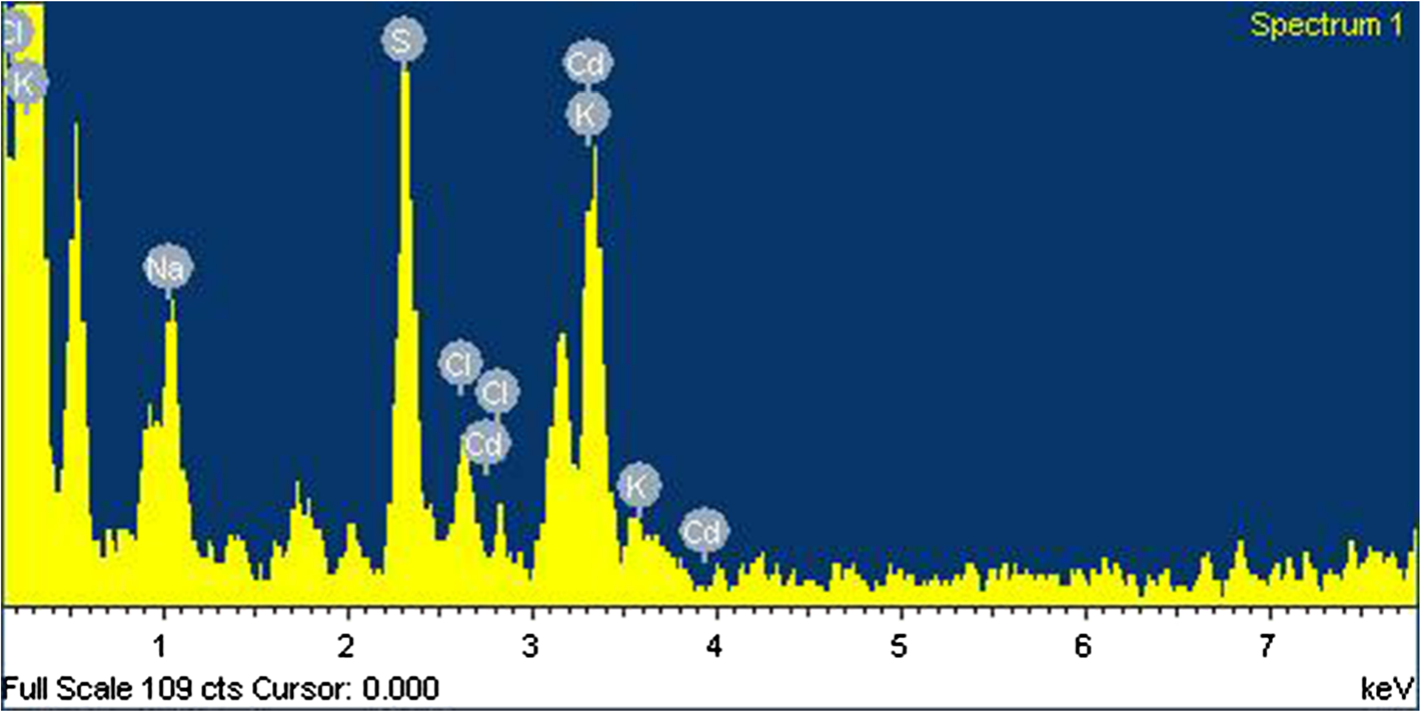
Elemental analysis of CdS quantum dots by X-ray emission spectroscopy

The HRTEM image (Fig. 4) confirmed the formation of CdS quantum dots. These particles are essentially spherical and they appear to be reasonably monodisperse. Their surface did not have any damages. The particle size histogram based on HRTEM micrographs is shown in Fig. 2. Total number of nanoparticles for particle size histogram accounted for 243 per field of view. One can see that the average particle ranges from 3.0 to 4.0 nm that corresponds to 50 % of nanoparticles, while nanoparticles 1.0 to 2.5 nm in diameter make up 7 %. The number of nanoparticles less than 4.5 nm in diameter does not exceed 15 %, and a small number of nanoparticles from 5 to 7 nm in diameter accounts for 5 % (Fig. 2). The electron diffraction patterns of cadmium sulfide quantum dots deposited on the carbon-coated copper grid are shown in Fig. 5. The diffraction maxima 1, 2, and 3 correspond to interplanar distances 0.334, 0.205, and 0.188 nm, indicating polycrystalline wurtzite modification [19]. The data obtained are identical to our first report on the CdS biosynthesis by fungi [11]. The results of an electron diffraction analysis in our recent research [12], in which we used a plant matrix for the CdS synthesis, were similar to the present data, and they proved the crystal lattice structure that was typical of a wurtzite modification in CdS nanoparticles. Moreover, in this study, we performed the elemental analysis of CdS samples, using the method of X-ray emission spectroscopy (Fig. 6). It has been found that Cd and S atoms prevailed in the samples of cadmium sulfide quantum dots. The presence of Na atoms can be explained as a byproduct of a chemical reaction, i.e., Na of Na_2_S salt was used in the CdS biosynthesis. The presence of K and Cl atoms was probably caused by the biological activity of components released from BY-2 cells. The biosynthesis of CdS nanoparticles was performed aseptically, all inorganic components were purified, and we therefore exclude the penetration of these ions from without. When comparing the present data with our previously published expanded study of the CdS synthesis by fungi, we can see that in [10], CdS quantum dots samples contained more additional elements such as atoms of oxygen, silicon, phosphorus, or iron. It may be due to the presence of macromolecular compounds secreted by mycelium. Additional inorganic components are an integral part of the CdS biosynthesis process, but it is better to minimize the presence of supplemental atoms, as they affect purity of the sample and may complicate the further applications of synthesized nanoparticles. In this research, one can see more homogenous samples of CdS quantum dots containing a small amount of other atoms, which do not affect their physical properties. Thus, the plant system is preferable for the CdS biosynthesis because it does not contain a large amount of the macromolecular compounds that affect the composition of the assay sample.

The present data of the HRTEM and electron diffraction analysis are similar to our previous investigations of the hairy root culture [12]. However, there were some differences in particle size distribution. In the research [12], we established that nanoparticles from 5.5 to 7 nm in diameter prevailed in the sample. In the present work, the sizes of particles are smaller, as they amount to 3.0–4.0 nm. Produced by the BY-2 culture, the samples of CdS quantum dots are more homogeneous as compared with [12]. The presence of smaller nanoparticles is worthwhile for bio-imaging applications because these nanoparticles infiltrate more easily through the cell membrane.

### Toxic Effect of Synthesized CdS Quantum Dots

**Fig. 7:**
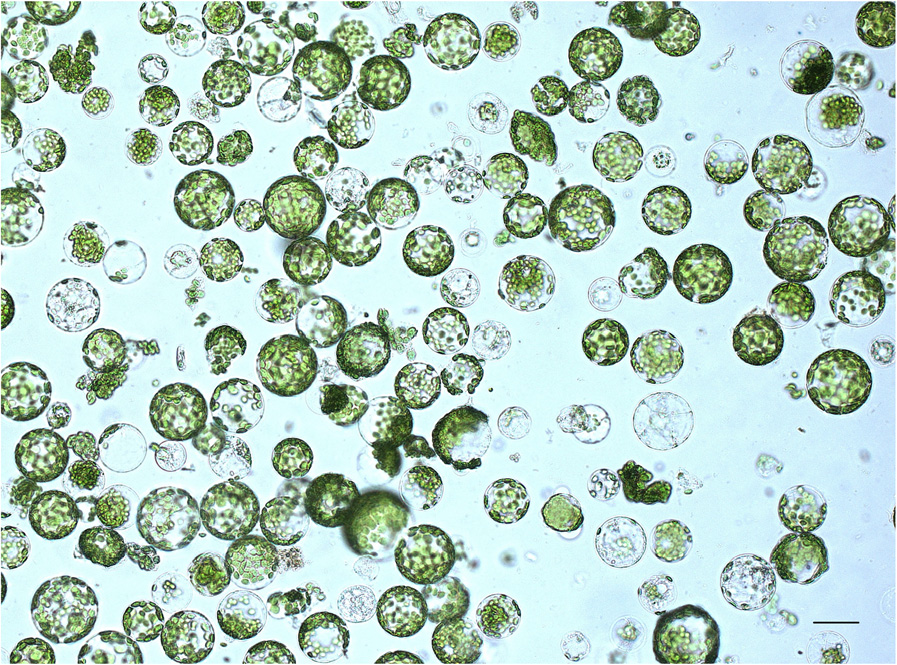
Freshly isolated tobacco protoplasts. *Scale bar* indicates 20 μm

**Fig. 8:**
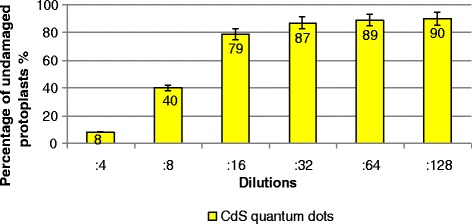
Toxic effect of CdS quantum dots

**Fig. 9:**
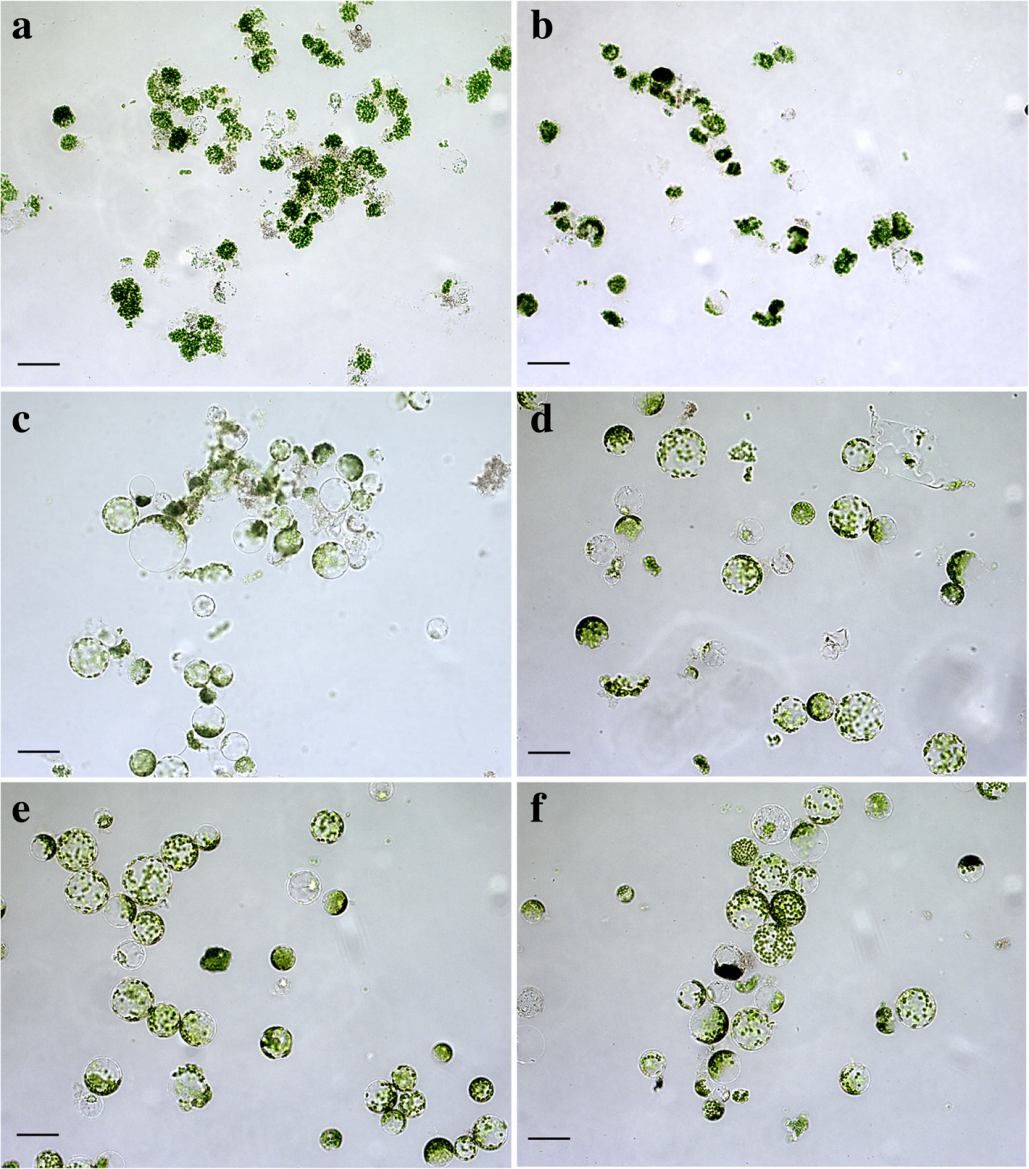
Protoplasts after incubation with different concentrations of CdS quantum dots. **a** 0.097 mg/mL. **b** 0.049 mg/mL. **c** 0.025 mg/mL. **d** 0.012 mg/mL. **e** 0.006 mg/mL. **f** 0.003 mg/mL. *Scale bar* indicates 20 μm

Protoplasts constitute the highly sensitive plant test system, which allows the biological compatibility of produced CdS quantum dots as well as their prospective usage in in vivo imaging of cells and subcellular structures. Plant protoplasts are the simplified, isolated models that have high sensitivity and demonstrate a rapid response to adverse external factors [20]. The absence of the cell wall is a key point for the induction of specific reactions of protoplasts to a potentially toxic factor. Freshly isolated *N. tabacum* protoplasts are shown in Fig. 7. The study of the toxic effect of CdS quantum dots on tobacco protoplasts demonstrated the absence of the negative influence of synthesized nanoparticles in low concentrations, namely 0.012 mg/mL (16-fold dilution), 0.006 mg/mL (32-fold dilution), 0.003 mg/mL (64-fold dilution), and 0.002 mg/mL (128-fold dilution) (Fig. 8). When plant protoplasts were treated with nanoparticles in higher concentrations—0.097 mg/mL (twofold dilution), 0.049 mg/mL (fourfold dilution), and 0.025 mg/mL (eightfold dilution), we observed an increase in a proportion of damaged protoplasts. Exposure of plant protoplasts with lower concentrations of CdS quantum dots did not affect their normal morphology as is clear from Fig. 9. Our research can be compared with the study [21] where authors tested toxic effects of chemically synthesized CdS nanoparticles on the aquatic plant *Spirodela polyrrhiza.* It has been found that *S. polyrrhiza* is particularly susceptible to CdS treatment at concentrations of up to 1 mg/L. In our research, biologically synthesized CdS quantum dots did not induce highly toxic effects on plant protoplasts. The concentrations ranging from 0.002 to 0.006 mg/mL did not have a negative influence on the morphological features and viability of *N. tabacum* protoplasts. Whereas in [21] CdS nanoparticles were synthesized by means of a hydrothermal method, they induced a profound toxic effect on the plant *S. polyrrhiza* even in low concentration (2 mg/L that corresponds to 0.002 mg/mL—the lowest concentration in our study). It may indicate the protective action of a biological coating around CdS quantum dots.

So, we can conclude that produced by the biological method, CdS quantum dots demonstrated the absence of a toxic effect on *N. tabacum* protoplasts in low concentrations. This result indicates that the biological synthesis of quantum dots decreases the toxic properties of cadmium, which agrees with [22].

The present work correlates with our recent studies in which we used biologically produced CdS quantum dots for the determination of their toxic effect on the living organism *Drosophila melanogaster* and cancer cells [23, 24]. In the case of an animal organism, CdS quantum dots synthesized by fungal and bacterial matrixes had the moderate toxic effect, which was much lower than that of ionic Cd. CdS nanoparticles did not produce genotoxic or mutagenic effects on *D. melanogaster* [23]. Different types of biologically synthesized CdS quantum dots were used for determining the toxic effect on cancer cells in [24]. We established that the cytostatic effect of CdS nanoparticles on HeLa cells decreased along with decreasing CdS concentrations. The effects on the adhesive potential of cells depended upon a type of CdS quantum dots.

The present study suggests a new approach to the CdS extracellular biosynthesis by the suspension culture of *N. tabacum* (BY-2)*.* The «green» method for producing cadmium sulfide nanoparticles has some advantages over previously used biological systems. In particular, the BY-2 suspension shows the rapid growth of a cell biomass, CdS samples are homogeneous (without precipitate), and obtained nanoparticles are water-soluble and mainly small-sized. The exact mechanisms of the formation of CdS quantum dots using plant cell suspension cultures has not been under study yet, but we can assume that the biosynthesis of glutathione, which is directly coupled to the uptake of SO_4_^2−^ ions in tobacco suspension cultures [25], or the secondary metabolites, which are secreted by BY-2 cells during incubation with appropriate inorganic compounds, could provide the formation of CdS quantum dots.

## Conclusions

This research is the first successful report on the extracellular biosynthesis of luminescent cadmium sulfide quantum dots using cell suspension culture of *N. tabacum* (cv. BY-2). The samples of CdS nanoparticles showed a clear absorption maximum at 292 that indicates the presence of small nanoparticles in the sample. A distinct luminescent peak at 381 nm was also found. According to HRTEM data, produced CdS quantum dots were either spherical or ellipsoid. Nanoparticles of 3–4 nm in diameter prevailed. It has been found that synthesized CdS nanoparticles in low concentrations do not exert any negative influence on the plant protoplasts. Hence, it appears that owing to their unique physical properties, the obtained CdS quantum dots may be promising for use as luminescent probes in bio-imaging studies. In addition, CdS quantum dots have a potential for being used in fluorescent labeling of proteins and cell tracking.
